# Does acute pancreatitis herald pancreatic ductal adenocarcinoma? A multicenter electronic health research network study

**DOI:** 10.1002/cam4.5094

**Published:** 2022-07-31

**Authors:** Ritu R. Singh, Alison P. Klein, Neil R. Sharma, Eileen M. O'Reilly

**Affiliations:** ^1^ Johns Hopkins School of Public Health Baltimore Maryland USA; ^2^ Parkview Health Fort Wayne Indiana USA; ^3^ Johns Hopkins School of Medicine Baltimore Maryland USA; ^4^ Memorial Sloan Kettering Cancer Center New York New York USA; ^5^ Weill Cornell Medical College New York New York USA

**Keywords:** acute pancreatitis, database, pancreas ductal adenocarcinoma, pancreatic neoplasm

## Abstract

**Background and Objectives:**

High mortality in pancreas ductal adenocarcinoma (PDAC) is related to delayed diagnosis and lack of cost‐effective early detection strategies. Retrospective studies have demonstrated an association between PDAC and acute pancreatitis (AP). Herein, we explore the incidence of PDAC in patients with non‐biliary and non‐alcoholic AP.

**Methods:**

A population‐based, retrospective cohort study was conducted utilizing TriNetX (Cambridge, MA). Patients ≥40 years with AP (ICD‐10‐CM code: K85) and without biliary AP (K85.1), alcohol‐induced AP (K85.2) or chronic pancreatitis (K86.0, K86.1), were identified. The primary outcome was incidence of PDAC (C25) in patients at defined intervals following AP. We compared the rate of early‐stage diagnosis (stage 1–2) and surgical resection among patients with and without preceding AP.

**Results:**

The incidence of PDAC ranged from 2.16% (1 year) to 3.43% (5 years). Patients with PDAC and AP in preceding year were more likely to undergo surgical resection relative to those without AP (10.1% vs. 6.3%, risk ratio 1.62: 95% confidence interval, CI 1.47–1.79). Early‐stage diagnosis of PDAC was more frequent in patients with preceding AP; however, difference was insignificant (*p* = 0.48; 95% CI 0.64–2.58).

**Conclusion:**

AP is infrequently associated with PDAC and can precede a diagnosis of PDAC in a minority of patients without another known etiology of pancreatitis. Patients with a recent AP are more likely to undergo surgical resection of PDAC and a trend toward diagnosis at an earlier stage compared to patients with PDAC and without AP. The impact of AP‐related PDAC on survival is unknown.

## Graphical abstract

Understanding the risk of pancreatic cancer following an acute pancreatitis diagnosis is a critical component to early intervention for pancreatic cancer in this high‐risk population as multiple data sets have demonstrated that early detection can lead to an increased percentage of patients diagnosed with stage I disease, and hence an improved chance for cure.

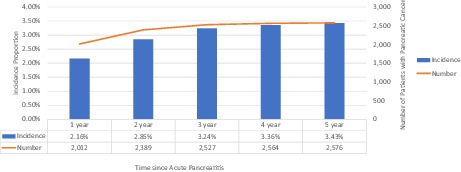

## 1 INTRODUCTION

Patients with pancreatic adenocarcinoma (PDAC) have the lowest 5‐year survival rate among all cancers and this rate has improved modestly from approximately 4% to 10% over the last 30 years.^1^ A longitudinal population‐based multinational study of seven common cancer sites comprising over 3.5 million patients identified PDAC to have the worst 5‐year survival rate of less than 10% in most countries.^2^ This lethality is in large part attributed to late clinical presentation, delay in diagnosis and frequent discovery of PDAC at unresectable and metastatic stages. Furthermore, it has been demonstrated that early diagnosis of PDAC is associated with higher probability of surgical resection and improved survival.^3^, ^4^

Several risk factors have been associated with PDAC, including chronic pancreatitis, tobacco smoking, selected inherited genetic variants, family history of PDAC, hereditary pancreatitis and possibly long‐standing diabetes mellitus.^5^, ^6^, ^7^, ^8^ Acute pancreatitis has been studied as a risk factor for PDAC as well as an associated clinical manifestation concurrent with the diagnosis of PDAC.^9^, ^10^, ^11^, ^12^ However, this has not been quantified adequately.^13^, ^14^

Acute pancreatitis can be an early sign of underlying PDAC. There are a few published retrospective, mostly population‐based studies evaluating the presence and predictive factors of underlying PDAC in patients presenting with acute pancreatitis.^11^, ^15^, ^16^, ^17^, ^18^ While there is some evidence to suggest that PDAC is diagnosed at an earlier stage in patients who present with PDAC during an episode of acute pancreatitis, there is limited prospective data regarding the clinical stage at diagnosis or survival outcomes.^11^, ^16^ Moreover, the frequency of PDAC detection is even higher among patients over 40 or 50 years of age who present with acute pancreatitis compared to those without pancreatitis.^18^, ^19^ The risk of identifying PDAC in these patients is higher in non‐biliary, non‐alcoholic etiologies of pancreatitis.^17^ Diagnosis at an early stage of PDAC (stages I–II) and subsequent surgical resection is associated with longer survival compared to individuals diagnosed with later stage disease.^3^, ^4^ Thus, exploring the hypothesis that diagnosing PDAC at an earlier stage in patients presenting with acute pancreatitis is a critical one, and herein we perform a retrospective cohort study utilizing a health research network based on electronic health records and insurance claims data.

We aim to determine the incidence of PDAC in patients with non‐biliary, non‐alcoholic acute pancreatitis at 3 months, and one through 5 years following a diagnosis of acute pancreatitis. Other objectives include determination of the rate of surgical resection and diagnosis of PDAC at an early stage in patients with and without a recent history of acute pancreatitis. We also aim to describe the anatomical location of PDAC (head, body/tail) in patients with acute pancreatitis, and evaluate the role of tumor markers (CA‐19‐9 and CEA) in predicting underlying PDAC in acute pancreatitis.

## 2 METHODS

This study is a population‐based, multi‐center, retrospective cohort study utilizing TriNetX (Cambridge, MA), “a global federated health research network that provides deidentified data from electronic medical records.” (https://www.trinetx.com/page/4/#home‐slider‐3‐copy) We searched the TriNetX platform to obtain aggregated health records from approximately 70 million patients in 55 health‐care organizations (HCO) from May 1, 2011, to April 30, 2021.

### 2.1 Study population

**FIGURE 1:**
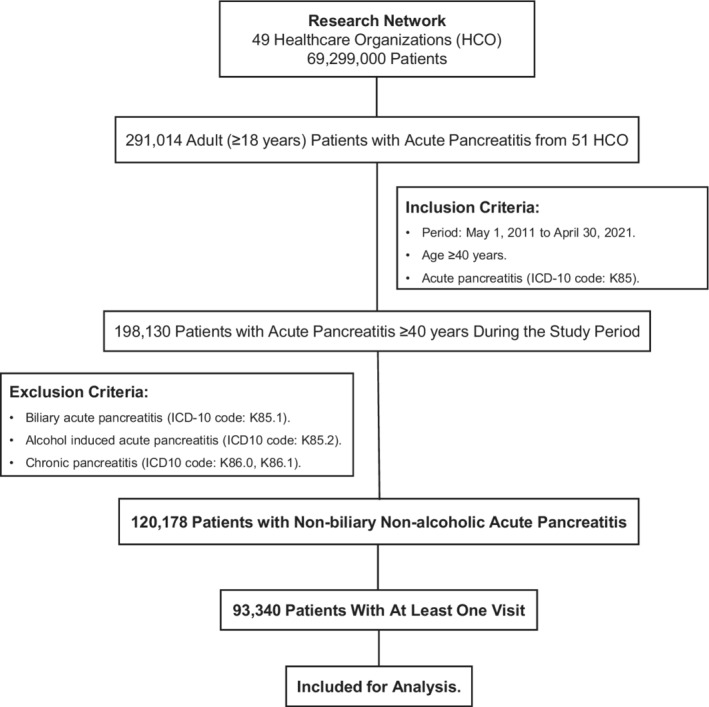
Consort flow diagram of database search and results.

Adult patients 40 years and older with a diagnosis of acute pancreatitis were identified using relevant ICD‐10‐CM codes (K85). Among these patients, those with biliary‐related acute pancreatitis (K85.1), alcohol‐induced acute pancreatitis (K85.2) and who had a diagnosis of chronic pancreatitis (ICD‐10‐CM codes: K86.0, K86.1), were excluded (Figure 1). This subgroup of patients with PDAC and acute pancreatitis was named acute pancreatitis of undetermined etiology (APUE). Patients with a diagnosis of exocrine PDAC were identified using appropriate ICD‐10‐CM codes (C25.0, C25.1, C25.2, C25.3, C25.7, C25.8, C25.9), and those with pancreatic endocrine neoplasms (ICD‐10‐CM code: C25.4), were excluded.

To determine the incidence of PDAC in APUE, we identified patients who had at least one visit (inpatient or ambulatory) during each consecutive year for 5 years following the diagnosis of acute pancreatitis. Patient cohorts were defined by time of acute pancreatitis diagnosis; acute pancreatitis diagnosed during May 2011–April 2013, May 2013–April 2015, May 2015–April 2017 and May 2017–April 2019.

In a separate analysis, a subset of patients who had a diagnosis of acute pancreatitis in the year preceding their diagnosis of PDAC were also examined for location of cancer within the pancreas (head, body, or tail), cancer stage and whether they underwent surgical resection. Patients presenting with acute pancreatitis a day or more following the diagnosis of PDAC were excluded.

### 2.2 Comparison group and matching

During the same period 2011–2021, patients with a diagnosis of PDAC and without a prior diagnosis of acute pancreatitis listed in the TriNetx dataset in the past were identified and selected as the matched comparison cohort. Patients in the two groups were matched for baseline characteristics including age, gender, race, common comorbidities (diabetes mellitus, obesity, malnutrition, chronic kidney disease, chronic pulmonary diseases, ischemic heart disease and heart failure), and visits (inpatient or ambulatory).

### 2.3 Follow up and clinical outcomes

The primary outcome of this study was to determine the incidence of PDAC following APUE in patients aged 40 years or older. We stratified the follow up period to evaluate the incidence rate at 3 months, and at yearly intervals for one through 5 years following the diagnosis of acute pancreatitis.

We analyzed both cohorts of PDAC patients (with and without preceding acute pancreatitis) for other outcomes, specifically the stage of PDAC at diagnosis and the rate of surgical resection in the first year following the diagnosis of PDAC. Early‐stage PDAC was defined as diagnosis of PDAC at stage I, stage IIa, or T1–T3 and N0. Surgical resection was defined as resection procedures performed in the first 12‐month period following the diagnosis of PDAC. Surgical resections were identified by CPT codes for distal subtotal pancreatectomy (1007918), proximal subtotal pancreatectomy with total duodenectomy or Whipple‐type procedure (1007923), pylorus‐sparing Whipple‐type procedure (1007926), total pancreatectomy (48155), near‐total pancreatectomy with preservation of duodenum (48146), and total or subtotal pancreatectomy with autologous islet cell transplantation (48160). We further identified surgical procedures through ICD‐10‐PCS codes, excision of pancreas via open approach (0FBG0ZZ), resection of pancreas via open approach (0FTG0ZZ).

We evaluated serum carbohydrate antigen 19‐9 (CA 19‐9) and serum carcinoembryonic antigen (CEA) values within a month of acute pancreatitis diagnosis in patients with and without PDAC. Laboratory codes 9055 (CA 19‐9) and 9056 (CEA) were used to identify the results. Abnormal CA 19–9 values were categorized into two groups, 37 to 100 units/ml and >100 units/ml. Patients with CEA values >5 ng/ml were identified in each cohort. We also evaluated the anatomical site of origin of PDAC where available in both cohorts of patients.

### 2.4 Statistical analyses

Mean and standard deviation were calculated for continuous variables, and proportion and percentage were calculated for dichotomous and categorical variables. Fisher's exact tests were used to compare characteristics (baseline and laboratory). Propensity score matching (1:1) was performed for baseline characteristics (age at the time of acute pancreatitis diagnosis, gender, race) and common morbidities (obesity, diabetes mellitus, chronic kidney disease, chronic pulmonary disease, heart failure, ischemic heart disease) using a ‘greedy nearest neighbor matching’ approach, and cohorts were considered well matched if there was a standardized mean difference of less than 0.1 for continuous variables. For clinical outcomes, risk ratio (RR) with 95% confidence interval (CI) and risk difference were calculated, and Kaplan‐Meir analysis with survival curve was obtained for primary outcomes.

### 2.5 Survival analysis

A life table was constructed to estimate the incidence of PDAC in patients with APUE (Tables S1 and S2). Incidence of PDAC in patients with APUE was calculated as a new diagnosis of PDAC within 3 months and each subsequent year for five consecutive years following an episode of acute pancreatitis. The denominator for this analysis was the total number of patients with APUE during each specified period. The statistical significance was set at 2‐sided *p*‐value of <0.05. All the statistical analyses were performed using the TriNetX platform.

### 2.6 Ethical considerations

This study involves human subjects; however, western institutional review board has provided a waiver to TriNetX as it utilizes aggregate counts and there is no access to protected health information from the participating HCO's. Thus, written patient consent is not required, nor feasible. Moreover, TriNetX rounds up number of patients to the nearest 10 for analytic purposes, so that the protected health information is fortified.^20^

## 3 RESULTS

**FIGURE 2:**
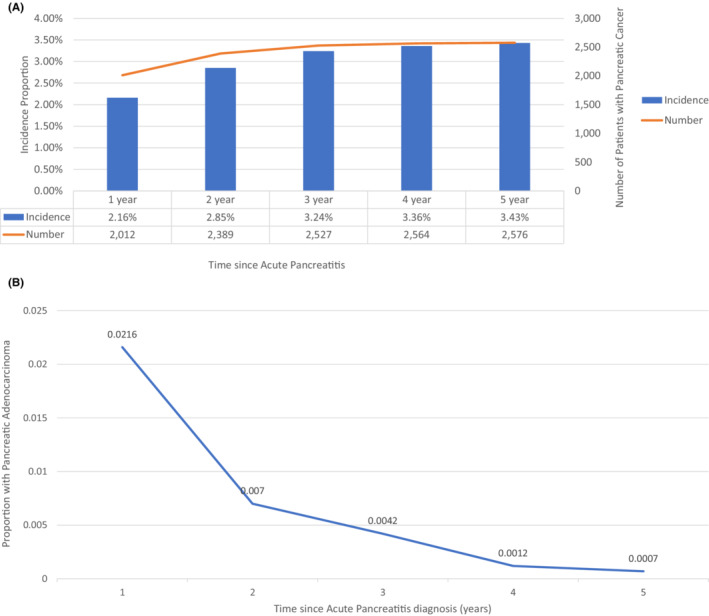
(A) Incidence of pancreatic adenocarcinoma in patients with acute pancreatitis. (B) Probability of developing pancreatic adenocarcinoma each year after acute pancreatitis.

There were 120,178 patients ≥40 years who were identified and diagnosed with APUE during the study period of 2011–2021. Of these, 93,340 had at least one visit in the year following acute pancreatitis diagnosis (Figure 1). The mean age of this cohort was 58.8 (±13.5) years at the time of diagnosis of acute pancreatitis and 51% were women. Incidence of PDAC was 1.78% at 3 months, 2.16% at 1 year, 3.24% at 3 years, and 3.43% at 5 years following acute pancreatitis (Figure 2A,B).

During the same period, 72,892 adult patients with PDAC were diagnosed from 48 HCO's. Patients with chronic pancreatitis and those with biliary, and alcohol‐related pancreatitis were excluded leaving 3902 (5.4%) patients who were diagnosed with acute pancreatitis in the year preceding the diagnosis of PDAC. Patients in the acute pancreatitis cohort were younger (65 years vs. 67 years, *p* < 0.001), and included more male patients (54% vs. 52%, *p* = 0.01) who were more likely to be obese (13.5% vs. 6.3%, *p* < 0.001) compared to PDAC patients without acute pancreatitis. Comorbidities including diabetes mellitus, chronic kidney disease, coronary artery disease, heart failure and chronic pulmonary diseases were more common in patients with acute pancreatitis compared to PDAC patients without acute pancreatitis. Approximately 9% of patients in the acute pancreatitis cohort had pancreatic cysts (excluding pseudocysts) compared to 3% of PDAC without acute pancreatitis (*p* < 0.001; Table 1).

**TABLE 1:** Baseline characteristics of patients with pancreas ductal adenocarcinoma

Demographics	Mean (±*SD*)		Number of patients (%)		*p*‐value
	AP	No AP	AP	No AP	
Age (years)	64.9 ± 12.2	66.9 ± 11.9	3902 (100%)	56,870 (100%)	<0.001
Female	—	—	1807 (46.3%)	27,493 (48.3%)	0.01
White race	—	—	2869 (73.5%)	21,388 (72.8%)	0.31
Diagnoses					
Diabetes mellitus			1009 (25.9%)	8187 (14.4%)	<0.001
Obesity			528 (13.5%)	3571 (6.3%)	<0.001
Chronic kidney disease			373 (9.6%)	2783 (4.9%)	<0.001
Pancreatic cyst			365 (9.3%)	1911 (3.4%)	<0.001
Ischemic heart disease			621 (15.9%)	4968 (8.7%)	<0.001
Heart failure			252 (6.5%)	2193 (3.9%)	<0.001
Chronic pulmonary disease			30 (0.8%)	182 (0.3%)	<0.001
Tobacco use			123 (3.2%)	784 (1.4%)	<0.001
Pancreatic cyst			365 (9.3%)	1911 (3.4%)	<0.001
Tumor markers					
Ca 19‐9					
37–100 unit/ml			274 (7.0%)	1054 (1.8%)	<0.001
>100 unit/ml			598 (15.3%)	3326 (5.8%)	<0.001
CEA >5 ng/ml			235 (6.0%)	1897 (3.3%)	<0.001

*Note*: Age, gender and all the listed diagnoses were matched.

Abbreviations: AP, acute pancreatitis; CA 19‐9 serum, carbohydrate antigen 19‐9; CEA serum, carcinoembryonic antigen; SD, standard deviation.

**FIGURE 3:**
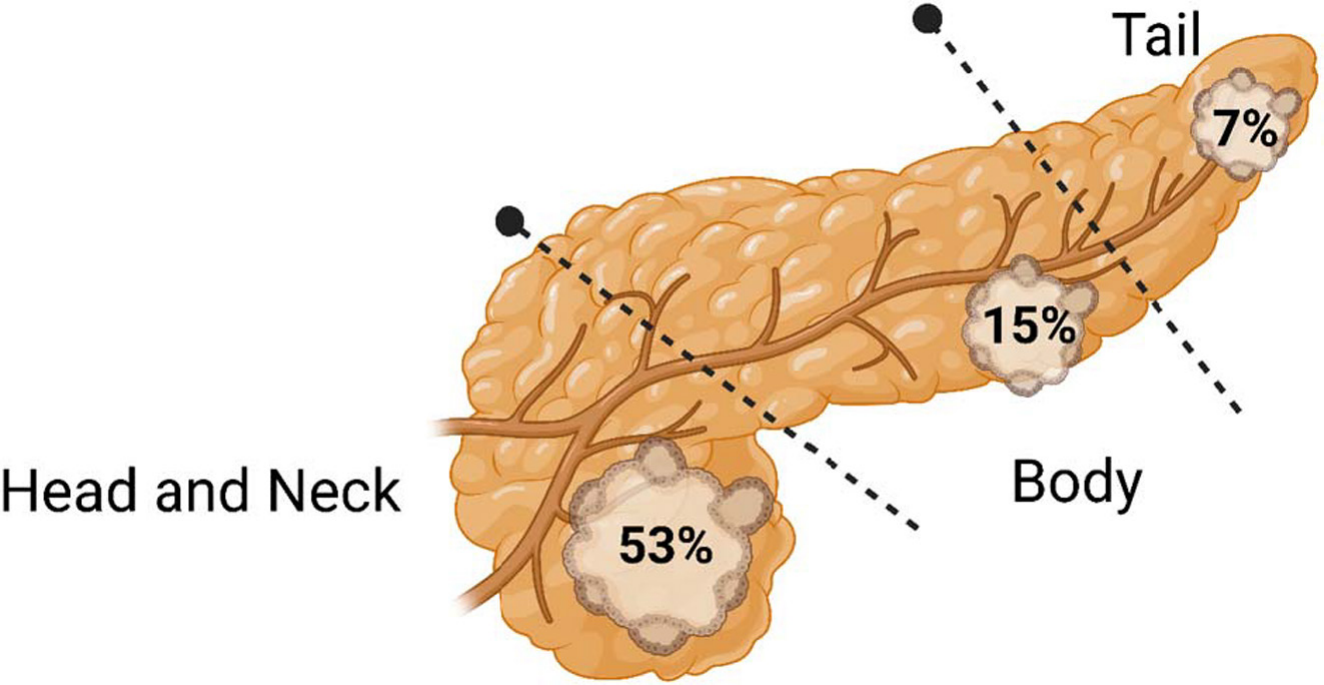
Image depicting location of pancreatic adenocarcinoma within the pancreas in patients with acute pancreatitis.

Patients with PDAC who had preceding acute pancreatitis underwent surgical resection more often than those who did not have a preceding diagnosis of acute pancreatitis (10.9% vs. 6.9%, risk ratio, odds ratio 1.58: 95% CI 1.37–1.82). There was a trend toward cancer detection at an earlier stage in patients with preceding acute pancreatitis compared to other patients with PDAC; however, this was not statistically significant (*p* = 0.48; 95% confidence interval 0.64–2.58) (Table 2). PDAC involved the pancreatic head in majority of patients with APUE (53%) while body (15%) and tail (7%) were less commonly involved (Figure 3). About 25% of patients did not have specified site of PDAC within the pancreas (overlapping areas of pancreas or unspecified location). Pancreatic head involvement was more common in patients with preceding acute pancreatitis compared to those without acute pancreatitis (53% vs. 27%, *p* < 0.001). Elevation of both tumor markers CA 19–9 (≥37 units/ml, >100 units/ml) and CEA (>5 ng/ml) was observed more frequently in the acute pancreatitis cohort compared to those without preceding acute pancreatitis. CA 19–9 (0.7% vs. 20%) and CEA (0.7% vs. 7%) were elevated in a much smaller proportion of patients with acute pancreatitis without PDAC compared to patients with acute pancreatitis and PDAC.

**TABLE 2:** Comparing patients with and without preceding pancreas ductal adenocarcinoma

Outcomes	PDAC with AP	PDAC without AP	Odds ratio (risk difference)	95% CI	*p* value
Surgical resection	10.9% (388)	6.9% (3504)	1.58 (3.8%)	1.37–1.82	<0.001
Early‐stage PDAC^a^	9.64% (376)	7.23% (4116)	1.3 (2.4%)	0.64–2.58	0.48
Tumor location^b^					
Head	53%	27.4%	—	—	<0.001

Abbreviations: AP, acute pancreatitis; CI, confidence interval; PDAC, pancreas ductal adenocarcinoma.

^a^ Stages I and IIa.

^b^ About 25% patients did not have specified location of cancer.

## 4 DISCUSSION

Efforts to identify a cost‐effective screening strategy to enable early diagnosis of PDAC can be offset by lack of clear causal factors or early clinical markers of the disease for most patients among other barriers. One of the major initiatives taken to enhance early detection of PDAC was creation of the Chronic Pancreatitis, Diabetes, and PDAC (CPDPC) Consortium with goals to establish large prospective cohorts of patients for longitudinal follow up.^21^ Thus far, a diagnosis of new‐onset diabetes within 3 years preceding a diagnosis of PDAC is one of the best described proximate clinical markers which may lead to the earlier detection of PDAC; however, the incidence of PDAC in this cohort was still only 1%–2% over a 3‐year period, and a previous study has reported even lower rate (<1%).^22^, ^23^ Thus, there is a need to continue pursuit of novel clinical indicators for early diagnosis of PDAC.

Available data suggest acute pancreatitis is a potential related factor for PDAC either as a presenting clinical manifestation or an etiological factor for its development.^11^, ^15^, ^16^ In this large electronic research network study, we observed a significantly increased risk of a PDAC diagnosis following an episode of APUE. The cumulative incidence of PDAC ranged from 2.2% by 1 year to 3.4% by 5 years following a diagnosis of APUE. While our results concur with previous data of an increased incidence of PDAC in the first few years following an episode of acute pancreatitis, strikingly, we observed that most of the diagnoses of PDAC occurred in the first 3 months to 1 year following an episode of acute pancreatitis.^9^, ^19^ Patients with PDAC and APUE were more likely to be younger, obese, and have an earlier stage‐specific PDAC diagnosis (10% vs. 7%), and more frequently underwent surgical resection (11% vs. 8%) compared to PDAC patients without acute pancreatitis. Overall, these findings are important and novel, and provide some key insights into the relationship between acute pancreatitis and PDAC.

While available data suggest an association of acute pancreatitis and PDAC in a significant minority of patients, the question remains whether acute pancreatitis is an etiologic risk factor for PDAC, or is it an early clinical manifestation of PDAC? Single‐center retrospective studies have reported that the risk of PDAC is highest in the year following acute pancreatitis followed by a rapid decrease in incidence over time.^12^, ^19^ Munigala et al. also observed that about 90% (69 of 76) of PDAC diagnoses following acute pancreatitis occurred in the first year. These observations agree with our conclusion that the highest incidence of PDAC (2.2%) occurs within the first year following a diagnosis of acute pancreatitis. Kirkegard et al. reported on a Danish registry to evaluate the risk of PDAC in patients hospitalized with acute pancreatitis, and over a 10 year follow up period. The risk of PDAC in individuals with acute pancreatitis was observed to be highest in the first 2 years, however, a significant 2‐fold risk increase was sustained for up to 10 years.^9^ Collectively these studies indicate the highest risk of PDAC is in the first 1–2 years following acute pancreatitis. This indicated that in many cases pancreatitis develops as a consequence of the developing cancer given brief time interval between the pancreatitis and cancer diagnosis. However, a prior history of pancreatitis, >2 years has also been reported more frequently in pancreatic cancer patients compared with controls.^12^ Furthermore, individuals with hereditary pancreatitis have a considerable risk of pancreatic cancer, indicating in some cases pancreatic cancer may arise due to underlying pancreatitis.

A retrospective Swedish cohort study followed patients with acute pancreatitis for up to 10 years and observed an increased incidence of PDAC compared to a non‐ acute pancreatitis cohort.^10^ Although they concluded that the risk was highest in the first 10 years after acute pancreatitis, approximately 60% of PDAC were diagnosed in the first year following acute pancreatitis. Similar observations were noted in a prior Swedish study where the risk of PDAC was highest in the first few years after acute pancreatitis and declined over the following years.^13^ Inflammation is an established risk factor for carcinogenesis. Some types of pancreatic cysts are known to be precursors of PDAC and may be missed, or not well characterized on routine cross‐sectional imaging.^24^, ^25^ Identification of high‐risk pancreatic cysts (intraductal papillary mucinous neoplasm and mucinous cystic adenomas) during evaluation of acute pancreatitis may provide an opportunity for heightened surveillance and early diagnosis at precancerous stage. Pancreatic cysts were detected more frequently in patients with a preceding diagnosis of acute pancreatitis (9.3% vs. 3.4%); however, there may be differences in the utilization of pancreatic imaging between the two groups of patients due to the presence or absence of acute pancreatitis. We examined the use of endoscopic ultrasound (EUS) for the diagnosis of PDAC and found that approximately 16% of patients with preceding AP had undergone EUS at the time of PDAC diagnosis compared to about 12% of patients without preceding AP. Due to lack of patient‐level data we cannot be certain which imaging modality was utilized for the diagnosis of PDAC at an individual patient level. Furthermore, more frequent use of EUS in AP cohort may have been related to management of local complications of AP.

Another important observation from our study was that head of pancreas cancer occurred approximately twice as commonly (53% vs. 27%) in patients who had preceding acute pancreatitis compared to those without acute pancreatitis. These findings are biologically intuitive and may be explained by the fact that PDAC in the pancreatic head can cause obstruction of the pancreatic duct leading to pancreatitis, and further supports the argument that acute pancreatitis is a clinical manifestation of PDAC rather than an etiologic entity in many cases.

Retrospective studies have identified that PDAC may be detected at an earlier stage in patients with a preceding diagnosis of acute pancreatitis.^11^, ^26^ A population‐based study comprising Danish and US (Medicare‐eligible) patients noted a lower frequency of metastatic disease, higher resection rate, and better survival in patients who had a diagnosis of acute pancreatitis within 90 days preceding PDAC.^16^ The findings are limited by the nature of the administrative database, and all patients with acute pancreatitis were included irrespective of etiology of acute pancreatitis.^16^ Nonetheless, these observations are in‐line with our results and indicate that an earlier diagnosis of PDAC leads to improved survival.

A key question remains as to whether there is a delay in the diagnosis of PDAC in patients who present with acute pancreatitis, and importantly if identification of PDAC is radiographically challenging in view of acute inflammatory changes, which may take several months to resolve. It is possible that a neoplastic mass may be mistakenly missed or occult due to these inflammatory changes, potentially delaying diagnosis.^10^ Nonetheless, the limited data available to date including the results herein suggest the contrary in that the diagnosis of PDAC occurs at an earlier stage and there is a higher probability of patients with PDAC and APUE able to undergo surgical resection compared to PDAC without APUE. We recommend very close follow up of patients with PDAC and APUE within the first few years given a 2%–3% risk of being diagnosed with PDAC in the subsequent 1 to 3 years following acute pancreatitis. Early use of more sensitive modalities such as endoscopic ultrasound may be warranted in patients with acute pancreatitis, especially APUE.

Investigation of isolated, asymptomatic elevation of CA 19‐9 commonly yields benign etiology, including AP, particularly, acute biliary pancreatitis, chronic pancreatitis and other hepatobiliary diseases.^27^, ^28^ We observed that both CA 19‐9 and CEA were more commonly elevated in patients with APUE and PDAC compared to PDAC patients without APUE. An interesting finding noted in our study is both CA19‐9 and CEA were rarely elevated (<1%) in patients with APUE in the absence of pancreatic adenocarcinoma. These findings may indicate an opportunity to utilize routine clinical biomarkers such as CA 19‐9 and CEA to enrich for possibility of underlying occult pancreatic adenocarcinoma in patients with APUE as there appears to be a low likelihood of confounding from acute pancreatitis per se. Moreover, the common etiology of elevation of CA 19‐9 in this setting, including acute biliary pancreatitis, and chronic pancreatitis, were excluded in both of cohorts. Retrospective studies have suggested an association between type 2 diabetes mellitus and elevated CEA and CA 19‐9.^29^, ^30^

Our study has several notable limitations. This study was designed as a retrospective cohort study and has an inherent risk of bias, both known and unknown. Although the database utilizes electronic health records for research purposes, detailed clinical information of individual patients is unavailable due to lack of access to protected health information. As with any other database, conversion of a patient's clinical data into codes can result in errors. TriNetX performs extensive data quality assessment to reduce the risk associated with data collection. Another limitation of using the EHR‐based database is the potential loss of patients if they transfer their care from one health network to another. For example, a patient with diagnosis of prior diagnosis of acute pancreatitis receives care for PDAC at a different health network. Furthermore, documentation of deceased patients may not be uniform, and the cause of death cannot be determined in individual patients. Strengths of our study include the recent time frame for study conduct (2011–2021), the inclusion of considerable number of healthcare organizations from different regions of the United States and abroad with access to approximately 70 million patient records, which collectively improves the generalizability of our results.

## 5 CONCLUSIONS

Acute pancreatitis is uncommonly associated with PDAC and can precede the diagnosis of PDAC in a considerable proportion of patients without another known etiology of pancreatitis. Patients with a recent episode of acute pancreatitis are more likely to have PDAC diagnosed at an earlier stage with a higher likelihood of undergoing surgical resection compared to patients without acute pancreatitis. CA 19‐9 and CEA can act as potential enrichment biomarkers of underlying PDAC in patients with APUE and can be utilized in conjunction with imaging modalities, including EUS, for surveillance of these patients given the increased risk of PDAC over the subsequent 1–3 years. Further prospective studies will inform the relationship between APUE and PDAC and evaluate whether APUE and PDAC confer a survival advantage relative to patients with PDAC and without APUE.

## AUTHOR CONTRIBUTIONS

Ritu R. Singh: Concept, design, analysis, and manuscript writing; Alison Klein: Design, manuscript writing, critical review, approval; Neil R. Sharma: Critical review and approval; Eileen M. O'Reilly: Design, manuscript writing, critical review, approval.

## FUNDING INFORMATION

Craig B. Thompson Cancer Center Support Grant P30 CA008748.

## CONFLICT OF INTEREST

Ritu R. Singh: None; Neil R. Sharma: Consultant for Boston Scientific, Medtronic, Mauna Kea, Steris medical; Eileen M. O'Reilly: Research Funding to MSK: Genentech/Roche, Celgene/BMS, BioNTech, AstraZeneca, Arcus, Elicio, Parker Institute; Consulting/DSMB: Cytomx Therapeutics (DSMB), Rafael Therapeutics (DSMB), Seagen, Boehringer Ingelheim, BioNTech, Ipsen, Merck, IDEAYA, Novartis, AstraZeneca, Noxxon, BioSapien, Novartis, Cend Therapeutics, Thetis, Autem, Agios (spouse), Genentech‐Roche (spouse), Eisai (spouse).

## ETHICS STATEMENT

This study involves human subjects; however, western institutional review board has provided a waiver to TriNetX since it utilizes aggregate counts and there is no access to protected health information from the participating HCO's.

## Supporting information


Appendix S1

## Data Availability

The data utilized for this study are available from TriNetX and restrictions apply to the availability of the data. Data can be made available at https://www.trinetx with the permission of TriNetX.
