# Supplementary material for: Does acute pancreatitis herald pancreatic ductal adenocarcinoma? A multicenter electronic health research network study
Source: Cancer Med. 2022 Jul 31;12(3):2505–13. doi: 10.1002/cam4.5094 (PMC9939170; doi:10.1002/cam4.5094)
Supplement: Supplementary file 1 — Appendix S1 [file CAM4-12-2505-s001.docx]

**Supplement table 1. Life table estimating the probability of pancreatic adenocarcinoma in patients with acute pancreatitis.**

| Since beginning of study period | AP at the beginning | PDAC during the period | Total PDAC at the end of each period | Lost to Follow up | At risk of PDAC | Proportion with PDAC during the period |
| --- | --- | --- | --- | --- | --- | --- |
| 1^st^ year | 93,340 | 2,012 | 2,012 | - | 93,340 | 0.2156 |
| 2^nd^ year | 91,328 | 377 | 2,389 | 37,582 | 72,537 | 0.0052 |
| 3^rd^ year | 53,369 | 138 | 2,527 | 19,801 | 43,468 | 0.0032 |
| 4^th^ year | 33,430 | 37 | 2,564 | 3,530 | 31,665 | 0.0011 |
| 5^th^ year | 29,863 | 12 | 2,576 | 12,599 | 23,563 | 0.0005 |

PDAC pancreatic adenocarcinoma, AP acute pancreatitis

**Supplement table 2.** **Life table Construction.**

| Years of AP diagnosis | AP 1-year FU | AP 2-year FU | AP 3-year FU | AP 4-year FU | AP 5-year FU | |  | PAC incidence | | | | |  | PAC at the end of year | | | | | |
| --- | --- | --- | --- | --- | --- | --- | --- | --- | --- | --- | --- | --- | --- | --- | --- | --- | --- | --- | --- |
|  |  |  |  |  |  |  | 1^st^ 3 months | 1^st^ year | 2^nd^ year | 3^rd^ year | 4^th^ year | 5^th^ year |  | 1^st^ year | 2^nd^ year | 3^rd^ year | 4^th^ year | 5^th^ year |  |
| 5/2011-4/2013 | 16,082 | 9,162 | 8,169 | 7,520 | 7,001 | | 207 (1.25%) | 246 (1.53%) | 57 (0.62%) | 37 (0.45%) | 23 (0.31%) | 12 (0.17%) |  | 15,836 | 9,105 | 8,132 | 7,497 | 6,989 |  |
| 5/2013-4/ 2015 | 22,577 | 13,580 | 12,257 | 11,255 | 10,263 | | 239 (1.04%) | 296 (1.31%) | 60 (0.44%) | 51 (0.41%) | 14 (0.12%) | 0 |  | 22,281 | 13,520 | 12,206 | 11,241 | 10263 |  |
| 5/2015-4/2017 | 24,198 | 14,692 | 13,142 | 11,125 | 8,451 | | 503 (2.05%) | 617 (2.55%) | 129 (0.88%) | 50 (0.38%) | 0 | - |  | 23,581 | 14,563 | 11,075 | 11,125 | 8,451 |  |
| 5/2017-4/2019 | 30,483 | 16,312 | 11,968 | 5,355 | 261 | | 711 (2.31%) | 853 (2.80%) | 131 (0.8%) | 10 | - | - |  | 29,630 | 16,181 | 11,958 | 5,355 | 261 |  |
| Total | 93,340 | 53,746 | 33,568 | 29,900 | 17,264 | | 1,660 (1.78%) | 2012 (2.16%) | 377 (0.70%) | 138 (0.41%) | 37 (0.12%) | 12 (0.07%) |  | 91,328 | 53,369 | 33,430 | 29,863 | 17,252 |  |
|  | | | | | | Follow up period | | | | | | | 3 months | 1^st^ year | 2^nd^ year | 3^rd^ year | 4^th^ year | 5^th^ year | |
|  | | | | | | Probability of surviving (not having Pancreatic cancer) | | | | | | | 98.23% | 97.84% | 99.30% | 99.59% | 99.88% | 99.93% | |
|  |  |  |  |  |  | Cumulative probability of having Pancreatic cancer | | | | | | | 1.78% | 2.16% | 2.85% | 3.24% | 3.36% | 3.43% | |

AP acute pancreatitis, FU follow up, PAC pancreatic adenocarcinoma
